# Doping with Multiscale Hybrid Particles Enhances the Thermal Conductivity and Insulation Properties of Epoxy Resin Composites

**DOI:** 10.3390/ma19091751

**Published:** 2026-04-24

**Authors:** Zhihui Xie, Yue Zhang, Mingpeng He, Yuanyuan Li, Menghan Wang, Cheng Xin, Zhipeng Lei

**Affiliations:** 1 Dongfang Electric Machinery Co., Ltd., Deyang 618000, China; xiezhihui123@126.com (Z.X.);; 2 Shanxi Key Laboratory of Mining Electrical Equipment and Intelligent Control, Taiyuan University of Technology, Taiyuan 030024, Chinaleizhipeng@tyut.edu.cn (Z.L.)

**Keywords:** epoxy resin, composites, thermal conductivity, insulating properties, finite element simulation

## Abstract

With the capacity of generators continuing to increase, higher demands are placed on the heat dissipation of epoxy resin (EP), the main insulation material used in stator bars and windings. To overcome its low thermal conductivity, a multiscale hybrid filler strategy was adopted to investigate the effects of spherical Al_2_O_3_ (10 and 1 μm), platelet BN (1 μm), and SiO_2_ (50 nm) on the thermal and insulating properties of EP composites. Unlike conventional studies focusing on individual fillers, this work highlights the synergistic design of fillers with different sizes and morphologies. The filler ratios were optimized by finite element simulation, and the composites were prepared by melt blending. The results show that, at a total filler loading of 38.5 wt%, the EP composite filled with spherical Al_2_O_3_ particles of 10 and 1 μm, platelet BN of 1 μm, and nano-SiO_2_ of 50 nm achieves a thermal conductivity of 0.5497 W/(m·K), corresponding to an increase of 158.2% compared with pure EP (0.2129 W/(m·K)). This enhancement is attributed to the synergistic effect of multiscale and multishape fillers, where large Al_2_O_3_ particles form the main thermally conductive framework, small Al_2_O_3_ particles fill the gaps, platelet BN acts as a bridging filler, and nano-SiO_2_ improves the interfacial region. In addition, the composite exhibits low relative permittivity and dissipation factor tan*δ* in the frequency range of 10^−2^–10^6^ Hz, and its breakdown strength reaches 65.99 kV/mm. These results demonstrate that simulation-guided multiscale hybrid filler design is an effective strategy for improving the thermal conductivity of EP while maintaining acceptable insulating performance.

## 1. Introduction

With the rapid development of generator technology towards high power and high energy density, the insulation and thermal stability of the stator coil’s main insulation materials are facing severe challenges. During the long-term operation of the motor, heat accumulation inside the main insulation causes local overheating, which accelerates the electrothermal aging of the insulation material, leading to the deterioration of insulation performance and the shortening of generator life [1,2,3,4,5]. Typical main insulation systems for generator stator bars and windings are composite structures composed of mica paper, glass cloth, and resin-based matrix phases [6]. Among them, epoxy resin is widely used as the key matrix and bonding phase because of its good processability, mechanical strength, and electrical insulation performance. However, the intrinsic thermal conductivity of epoxy resin is only about 0.2 W/(m·K), which severely limits heat dissipation inside the motor and becomes a key factor restricting the improvement of generator cooling efficiency and long-term stable operation [7]. Traditional thermal-management strategies mainly rely on optimizing external cooling structures, such as ventilation ducts, airflow paths, and auxiliary cooling channels, to reduce the temperature rise in electrical machines [8,9]. However, these approaches often involve more complex structural design, additional cooling components, and larger installation space, which increase equipment volume and manufacturing cost. Therefore, thermal conductivity modification of the epoxy resin matrix has become an effective solution. By improving the thermal conductivity of the material, the heat dissipation performance of the motor insulation system is essentially improved, thereby achieving the unity of efficient heat dissipation and long-term reliable operation while maintaining the advantages in equipment volume and costs.

The thermal conduction of epoxy resin relies on phonons rather than free electrons. However, the disordered entanglement of molecular chains, low crystallinity, and polydispersity of molecular weight give rise to intense phonon scattering, resulting in low intrinsic thermal conductivity [10]. At present, two approaches are available to improve the thermal conductivity of epoxy resin: the intrinsic method and the filling method. The intrinsic method suppresses phonon scattering by regulating the chain structure through molecular engineering to achieve an essential improvement in thermal conductivity [11]. The filling method improves thermal conductivity by adding high thermal conductivity inorganic fillers to blend with the matrix and leveraging the synergistic heat transfer effect of the fillers. Due to the complex process, high cost, and great difficulty in practical engineering implementation of the intrinsic method [12], solving the problem of a low thermal conductivity of epoxy resin focuses more on the filling method.

At present, high thermal conductivity inorganic fillers commonly used for doping into epoxy matrices include Al_2_O_3_, BN, AlN, SiO_2_, etc. Compared with metals and carbon-based materials, these fillers generally exhibit better electrical insulation, making them more suitable for high-voltage power equipment [13]. Although single-filler incorporation can improve the thermal conductivity of epoxy resin, its enhancement is still limited, and it is often difficult to simultaneously achieve high thermal conductivity and good electrical insulation [14]. Therefore, recent studies have increasingly focused on hybrid-filler design, filler-size optimization, and interparticle-bridging strategies to construct more efficient heat-conduction pathways.

For example, Wang et al. used BN and MWCNTs to construct continuous heat-conduction pathways in epoxy resin, and achieved a thermal conductivity of 0.92 W/(m·K) at a BN/MWCNT ratio of 15:1 and a total filler loading of 30 wt%, corresponding to a 118% increase over neat epoxy [15]. Wang Wei et al. further demonstrated that, by in situ forming silver-nanoparticle bridges between Al_2_O_3_ microspheres, the thermal conductivity could be increased from 1.38 to 2.62 W/(m·K) at 60 vol% Al_2_O_3_, while the volume resistivity and dielectric strength were largely maintained [16]. In addition, Pawelski-Hoell et al. showed that filler-size selection strongly affected thermal-network formation; when 12 μm platelet BN was combined with 2 μm boehmite, the through-plane thermal conductivity increased from approximately 0.2 to 1.04 W/(m·K) [17]. These studies indicate that the rational combination of filler size, morphology, and interparticle connectivity is effective for enhancing heat transport in epoxy composites. However, although higher filler loading and multiscale filler design can significantly enhance thermal conductivity, they may also compromise dielectric properties and processability. Feng et al. prepared multi-scale Al_2_O_3_/EP composites by adding 5 μm Al_2_O_3_ (26.67 vol%), 30 μm Al_2_O_3_ (27.41 vol%), and 70 μm Al_2_O_3_ (45.92 vol%) to epoxy resin. The thermal conductivity reached 2.707 W/(m·K), which is about 12 times higher than that of pure EP. However, its relative permittivity increased to 6.0–7.0, the dielectric performance deteriorated, and the viscosity of the uncured mixture increased significantly, which would adversely affect the processability of the composite [18]. Yao Tong et al. prepared micro-nano binary filler-filled epoxy resin through BN and Al_2_O_3_, and found that the thermal conductivity of the epoxy resin composite with 22.5 wt%BN and 7.5 wt%Al_2_O_3_ can reach 1.35 W/(m·K). The dielectric loss is higher than that of pure EP, but the electrical performance of the material decreases [19]. Strauchs et al. reported that, in epoxy-based syntactic foam, adding 2.5 wt% and 5 wt%nano-SiO_2_ slightly increased the permittivity from 2.59 to 2.66 and 2.70, respectively, and increased the loss factor from 7.0 × 10^−3^ to 8.3 × 10^−3^ and 8.5 × 10^−3^, while the volume resistivity remained at 0.5 × 10^15^ Ω·m and the breakdown strength showed no significant change [20]. By contrast, Li et al. reported that low-content nano-SiO_2_ could improve the electrical performance of epoxy resin: the relative permittivity decreased from 3.1 for neat EP to 2.3 and 2.6 for 0.5 wt%SiO_2_-EP and 1 wt%SiO_2_-EP, respectively, the tree inception voltage of 1 wt%SiO_2_-EP was on average 24% higher than that of pure EP, and the average breakdown strength of 0.5 wt%SiO_2_-EP and 1 wt%SiO_2_-EP increased by 5.8% and 4.0%, respectively [21].

This work aims to develop a multiscale and multishape hybrid filler strategy for epoxy resin composites with improved thermal conductivity and acceptable insulating performance. Platelet BN, spherical Al_2_O_3_ particles with different sizes, and nano-SiO_2_ were combined according to their functional roles to construct thermally conductive pathways and regulate the interfacial region. Finite element simulation was first employed to optimize the filler formulation, followed by the preparation of epoxy composites with different compositions. The thermal conductivity, dielectric spectra, and breakdown strength of the samples were then measured. By combining simulation with experiment, the synergistic effects of multiscale fillers on heat-conduction-path construction and insulation performance were systematically analyzed.

## 2. Sample Preparation and Experimental Methods

### 2.1. Experimental Raw Materials

Epoxy resin: Bisphenol A diglycidyl ether (DGEBA), supplied by Dow Chemical Company (Midland, MI, USA), model DER332, with an epoxy equivalent of 171–175 g/ep and a viscosity (25 °C) of 4000–6000 cP; Curing agent: Polyetheramine D-230, purchased from Shanghai Aladdin Biochemical Technology Co., Ltd. (Shanghai, China); Platelet BN: Hexagonal flake structure, with average particle sizes of 50 nm and 1 μm, purchased from Shanghai Aladdin Biochemical Technology Co., Ltd. (Shanghai, China) and Beijing Deke Daojin Technology Co., Ltd. (Beijing, China), respectively; Aluminum oxide (Al_2_O_3_): Spherical structure, with average particle sizes of 10 μm and 1 μm, purchased from Beijing Deke Daojin Technology Co., Ltd.; Silicon dioxide (SiO_2_): Spherical structure, with an average particle size of 30 nm, purchased from Shanghai Zhiji Biochemical Technology Co., Ltd. (Shanghai, China).

### 2.2. Sample Preparation

**Figure 1:**
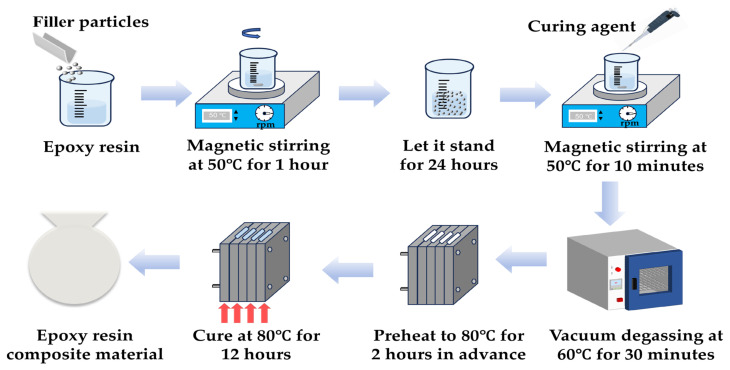
Composite epoxy resin preparation process.

Epoxy composite samples with different group designations and compositions, as summarized in Table 1, were prepared by melt blending under elevated temperature to reduce resin viscosity and promote uniform filler dispersion, as illustrated in Figure 1.

Firstly, the filler particles were dried at 80 °C for 24 h, and the epoxy resin was preheated at 80 °C for 15–20 min to reduce its viscosity. Secondly, an appropriate amount of EP and the corresponding mass fraction of particles were weighed and mixed. After mixing, mechanical stirring was used, and stirring was maintained at 800 rpm for 1 h at 50 °C. After stirring, the mixture was left at room temperature for 24 h to obtain the first mixture. Subsequently, before formal pouring, the mold for preparing epoxy resin samples was preheated at 80 °C for 2 h; then the curing agent was added to the first mixed solution (the mass ratio of curing agent to EP was 1:3) and mechanically stirred for 10 min. Finally, the resulting mixture was degassed in a vacuum oven at 60 °C for 30 min before being poured into the preheated mold. The sample was then cured at 80 °C for 12 h, followed by cooling at room temperature for 6 h before demolding.

### 2.3. Characterization and Simulation Methods

#### 2.3.1. Finite Element Modeling

Digimat 2017 was used to establish a three-dimensional random Representative Volume Element (RVE) model of the composite to simulate the distribution of filler particles within the epoxy matrix and to predict the overall thermal conductivity of the composite. The established model was imported into COMSOL Multiphysics 6.1, where the corresponding material parameters and boundary conditions were assigned. Subsequently, mesh generation was performed to enable the numerical calculations. In the thermal conductivity simulation, it was assumed that the thermal interfacial resistance between the fillers and the matrix was negligible, meaning that the interface was considered to be in ideal thermal contact. In this paper, the Heat Transfer in Solids module was used to simulate the thermal conductivity under steady-state conditions. Epoxy resin was used as the matrix, and Al_2_O_3_, BN, and SiO_2_ were used as fillers. The detailed parameter settings of the aforementioned materials are summarized in Table 2.

In this simulation, the thermal conductivities of all fillers were treated as constant bulk values. Size-dependent effects were not explicitly considered, as the aim of this study was to evaluate the relative influence of different filler combinations on the effective thermal conductivity of the composites rather than to establish a nanoscale phonon transport model.

The boundary conditions of the model were carefully defined. Specifically, the *Z*-axis was designated as the heat transfer direction. The upper boundary was defined as a high-temperature boundary with a temperature of 293.15 K + dT, whereas the lower boundary was set as a low-temperature boundary at 293.15 K. Herein, dT denotes the applied temperature difference, which was fixed at 50 K in the present work. To minimize external thermal interference, the other four surfaces were set as adiabatic boundaries with no heat exchange with the external environment. Subsequently, the Fourier equation was used to calculate the thermal conductivity of the model, with the low-temperature boundary as the calculation object. The calculation formula is given as follows:

$$\lambda =-q/\frac{dT}{dx}$$

where λ is the thermal conductivity; q is the heat flux density; $\frac{dT}{dx}$ is the temperature gradient.

The heat flux density through the surface was obtained by integrating the heat on the low-temperature boundary. The formula was defined in the parameter settings of COMSOL for non-local coupling calculation to obtain the simulated thermal conductivity value. A steady-state study was conducted on the model.

For electric-field simulation, the Electrostatics module in COMSOL was used. The electric field was applied along the *Z*-axis direction. For the 30 μm and 1 μm RVE models, voltages of 1.05 kV and 35 V were applied to the upper surfaces, respectively, while the lower surfaces were grounded, thereby maintaining an average electric-field strength of 35 kV/mm in both models. This value corresponds to the typical operating electric field of generator stator windings [22]. The relative permittivity values of the epoxy matrix and fillers are listed in Table 2.

To quantitatively evaluate the electric-field distortion, the electric-field enhancement factor, denoted as β, was introduced and defined as:

$$\beta =E_{\max}/E_{0}$$

where $E_{\max}$ is the maximum local electric field, and $E_{0}$ is the applied average electric field.

It should be noted that the simulation cases were constructed for numerical analysis and were not intended to exactly reproduce all experimental formulations. While the single-filler systems were kept consistent with the experimental groups for comparison and model validation, the hybrid simulation cases were simplified because micron-sized fillers and nano-sized fillers could not be simultaneously incorporated into the present RVE model with acceptable computational feasibility and statistical representativeness. Therefore, the simulation cases mainly represent the simplified numerical systems used for mechanism analysis, rather than all experimentally prepared formulations.

#### 2.3.2. SEM Observation

The cross-sectional microstructure of the composite was observed using a ZEISS Gemini 300 scanning electron microscope (SEM, ZEISS, Oberkochen, Germany).

#### 2.3.3. Thermal Conductivity Measurement

A HotDisk TPS2500S thermal conductivity analyzer (HotDisk, Gothenburg, Sweden) was used to conduct the measurements. The thermal conductivity test range of the analyzer was 0.005 W/(m·K) to 500 W/(m·K), with a measurement accuracy of ±3%. The required samples were two groups of square specimens with dimensions of 20 mm × 20 mm × 3 mm.

#### 2.3.4. Dielectric Performance Measurement

A Novocontrol Concept80 broadband dielectric spectrometer (Novocontrol, Montabaur, Germany) was used to perform the measurements. An alternating current (AC) voltage of 3 V was applied to measure the relative permittivity and dielectric loss of the samples under test in the frequency range of 10^−2^ Hz to 10^6^ Hz.

#### 2.3.5. Breakdown Field Strength Measurement

**Figure 2:**
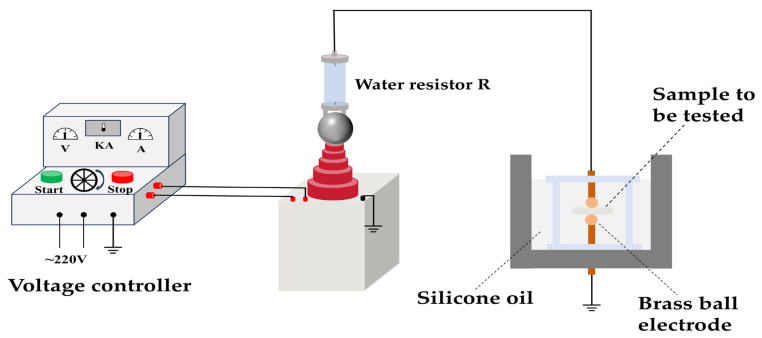
Breakdown field strength test circuit.

Measurements were conducted using a laboratory self-built experimental platform, as shown in Figure 2. Ball-ball electrodes were used in this study. All samples were disc-shaped with a diameter of 10 mm and a thickness of 0.3 mm. At power frequency, the voltage was increased stepwise every 20 s. The initial voltage was set to 2 kV, and the voltage was increased by 1 kV at 20 s intervals until the samples under test broke down. To eliminate randomness, ten measurement points were taken for each group of samples.

## 3. Simulation Results

Based on the finite element modeling procedure described in Section 2.3.1, the simulation results of thermal conductivity and electric-field distribution are presented in this section. These results were used to evaluate the effects of different filler types, particle sizes, and hybrid configurations, and to guide the subsequent experimental formulation design.

### 3.1. Thermal Conductivity Simulation

**Figure 3:**
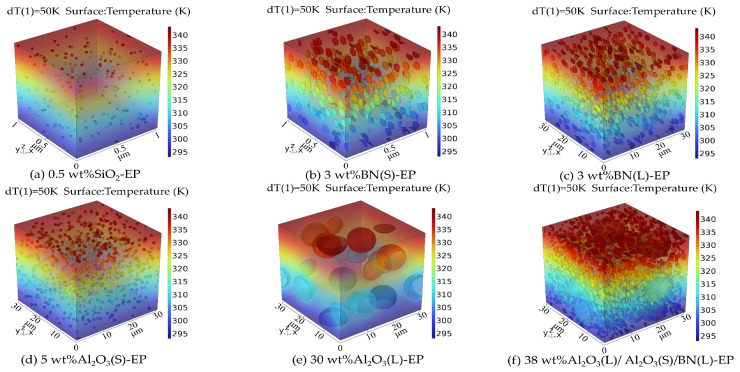
Cloud diagram of temperature distribution of epoxy resin composites in each group.

**Figure 4:**
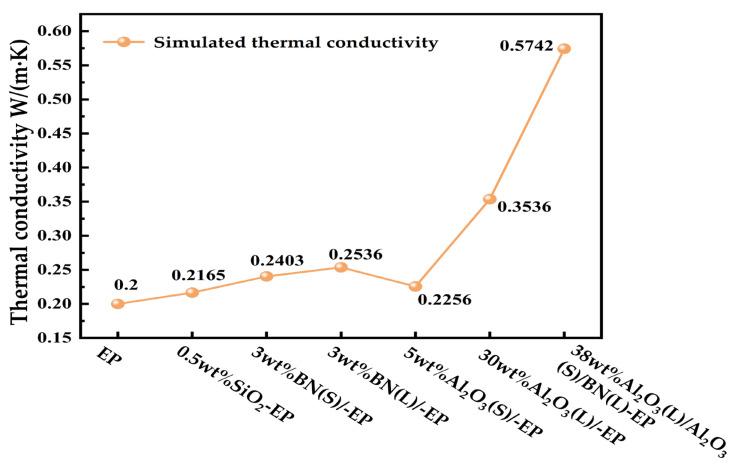
Thermal conductivity simulation values of epoxy resin composites.

Figure 3 shows the simulated temperature-distribution cloud maps of the different epoxy composite systems, and Figure 4 summarizes the corresponding effective thermal conductivity values. In total, seven simulation cases were considered, including both single-filler systems and hybrid filler systems. The particle types, particle sizes, mass fractions, and sample designations of the simulation cases are summarized in Table 3. Herein, Al_2_O_3_(L), Al_2_O_3_(S), BN(L), and BN(S) denote particles with characteristic sizes of 10 μm, 1 μm, 1 μm, and 50 nm, respectively.

As shown in Figure 4, the incorporation of thermally conductive fillers generally increases the effective thermal conductivity of epoxy resin, although the extent of improvement depends strongly on filler type, particle size, and hybrid structure. The 0.5 wt%SiO_2_-EP system exhibits the lowest simulated thermal conductivity among the filled systems, at 0.2165 W/(m·K), which is only slightly higher than that of neat EP. Under single-filler conditions, the simulated thermal conductivity values of 3 wt%BN(S)-EP, 3 wt%BN(L)-EP, and 5 wt%Al_2_O_3_(S)-EP are 0.2403, 0.2536, and 0.2256 W/(m·K), respectively. Among these systems, BN provides a more pronounced improvement in thermal conductivity. In addition, the larger BN(L) particles exhibit a higher simulated thermal conductivity than BN(S), indicating an evident particle-size effect. The hybrid system 38 wt%Al_2_O_3_(L)/Al_2_O_3_(S)/BN(L)-EP shows the highest simulated thermal conductivity, reaching 0.5742 W/(m·K), which is 187.1% higher than that of neat EP.

To further evaluate the robustness of the model, a sensitivity analysis was conducted by varying the thermal conductivity values of BN, Al_2_O_3_, and SiO_2_ within ±20% of the reference values in Table 1. The results show that the predicted effective thermal conductivity changes only slightly, indicating that the model is reasonably robust with respect to uncertainties in filler thermal conductivity. The detailed results are summarized in Table 4.

### 3.2. Electric Field Distribution Simulation

**Figure 5:**
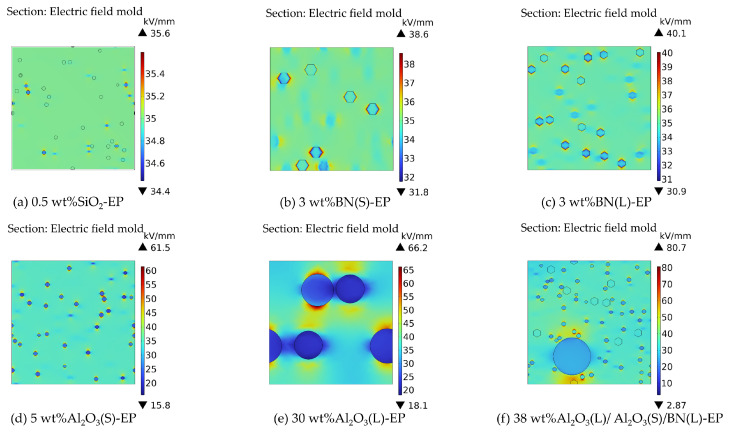
Cloud map of electric field distribution of epoxy resin composites in each group.

**Figure 6:**
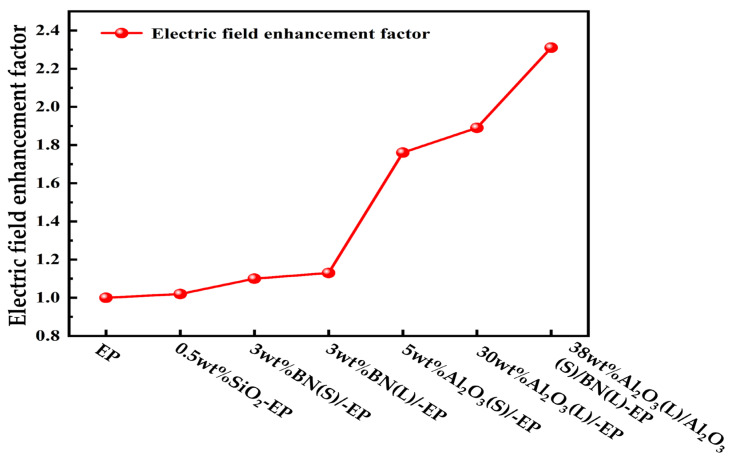
Electric-field enhancement factor (β) of different epoxy composite systems.

Figure 5 presents the simulated electric-field distributions of the different composite systems, and Figure 6 summarizes the corresponding electric-field enhancement factors. To facilitate observation, a cross-section of each three-dimensional model was selected for analysis. The results show clear differences in field uniformity among the investigated filler systems, indicating that filler type, particle size, and hybridization strategy all affect local electric-field distortion within the epoxy matrix. Among the investigated systems, the 0.5 wt%SiO_2_-EP composite exhibits the most uniform electric-field distribution and the lowest local field distortion. By contrast, the hybrid system 38 wt%Al_2_O_3_(L)/Al_2_O_3_(S)/BN(L)-EP exhibits the most pronounced field non-uniformity and the highest local field concentration. Under identical conditions, the maximum distorted field values of BN(S)-EP and BN(L)-EP are 38.6 kV/mm and 40.1 kV/mm, respectively, indicating that smaller BN particles lead to relatively lower electric-field distortion.

As shown in Figure 6, neat EP exhibits a β value of 1.00, corresponding to an almost perfectly uniform electric-field distribution. Among the filled systems, the 0.5 wt%SiO_2_-EP composite shows the lowest β value of 1.02, whereas the hybrid composite 38 wt%Al_2_O_3_(L)/Al_2_O_3_(S)/BN(L)-EP exhibits the highest β value of 2.31. These results indicate that high filler loading and multi-particle interactions intensify local electric-field concentration in the composite.

Based on the combined simulation results of thermal conductivity and electric-field distribution, the Al_2_O_3_(L)/Al_2_O_3_(S)/BN(L)-EP hybrid system was identified as a promising thermally conductive framework, but its electric-field distortion was relatively severe. Therefore, in the subsequent experimental design, low-content nano-SiO_2_ was further introduced to improve the interfacial region and mitigate electrical-performance deterioration. It should be noted that no direct multiscale simulation was performed for the final hybrid formulations containing both micron-sized fillers and nano-SiO_2_, because these two scales could not be simultaneously incorporated into the same RVE model in the present simulation framework.

Based on the systematic results of thermal conductivity and electric field distribution simulations, the thermal conductivity of the Al_2_O_3_(L)/Al_2_O_3_(S)/BN(L)-EP composite is significantly improved, whereas its electric field distribution is uneven, leading to significant electric field distortion. However, the low electric field at the SiO_2_ particle/EP interface can effectively mitigate the overall electric field distortion. Adding SiO_2_ can greatly eliminate the negative impact of Al_2_O_3_ on the electrical insulation performance, thereby reducing the electric field distortion. In view of this, in the subsequent experimental tests, 0.5 wt%nano-SiO_2_ was added to Al_2_O_3_(L)/Al_2_O_3_(S)/BN(L)-EP, and the thermal conductivity and electrical performance were tested. In the COMSOL multiphysics simulations employed in this study, micron-sized particles and nanoparticles cannot be incorporated into the same unit cell model. Therefore, the Al_2_O_3_(L)/Al_2_O_3_(S)/BN(S)-EP composite samples will be fabricated and characterized for comparison in subsequent work.

## 4. Experimental Results and Discussion

Before discussing the detailed experimental results, it should be emphasized that the studied systems include both single-filler and hybrid-filler epoxy composites. Finite element simulations had previously been conducted to obtain a preliminary understanding of how the incorporation of single fillers and multiscale hybrid fillers influences the thermal and electrical properties of epoxy resin. Based on these simulation results, the hybrid formulations were designed according to the size scale, morphology, and expected functions of the fillers: large spherical Al_2_O_3_ particles were used to construct the primary thermally conductive framework, small spherical Al_2_O_3_ particles were introduced to fill the interparticle gaps, platelet BN was incorporated to bridge adjacent particles and enhance heat transfer, and nano-SiO_2_ was added to regulate the interfacial region and improve insulation performance. Therefore, the following results should be understood not only as a comparison among different composite systems, but more importantly as an experimental verification and further evaluation of the proposed multiscale and multishape hybrid design concept.

### 4.1. SEM Characterization

**Figure 7:**
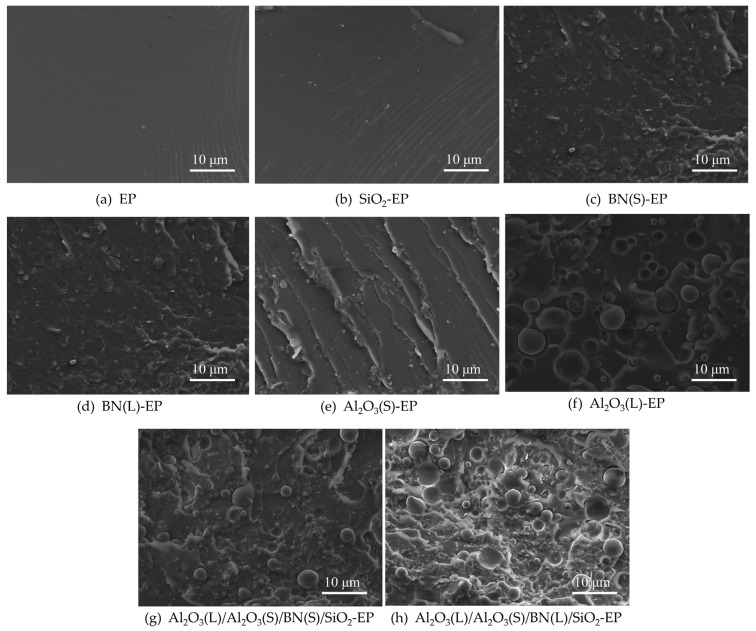
SEM images of epoxy resin composite materials from each group.

Figure 7 shows the micromorphology of the epoxy resin composites. It can be observed from Figure 7a that the brittle fracture surface of neat EP is flat and smooth without internal defects. The white bright spots in Figure 7b correspond to SiO_2_. It can be observed from the figure that the nanoparticles are relatively uniformly dispersed in the epoxy resin matrix, indicating that the composite sample has been successfully prepared. Figure 7c,d exhibits uneven brittle fracture surfaces, which arise from the shear yielding of the EP matrix induced by BN particles. The platelet BN particles are dispersed in the matrix in an island-like phase structure. It can be observed from Figure 7f that the spherical Al_2_O_3_ particles are uniformly dispersed in the matrix, and the dark pits result from the detachment of Al_2_O_3_ particles during the low-temperature brittle fracture. Although the Platelet BN particles exhibit some degree of preferential orientation, they are generally well-dispersed in the matrix without forming large agglomerates. This dispersion state, combined with the “sphere-flake” structure formed with Al_2_O_3_ particles, contributes to the formation of efficient thermal conduction pathways.

### 4.2. Thermal Conductivity

#### 4.2.1. Thermal Conductivity Results

The thermal conductivity of the epoxy composite samples was measured at room temperature. Each sample was tested three times, and the average value was taken as the final thermal conductivity result.

**Figure 8:**
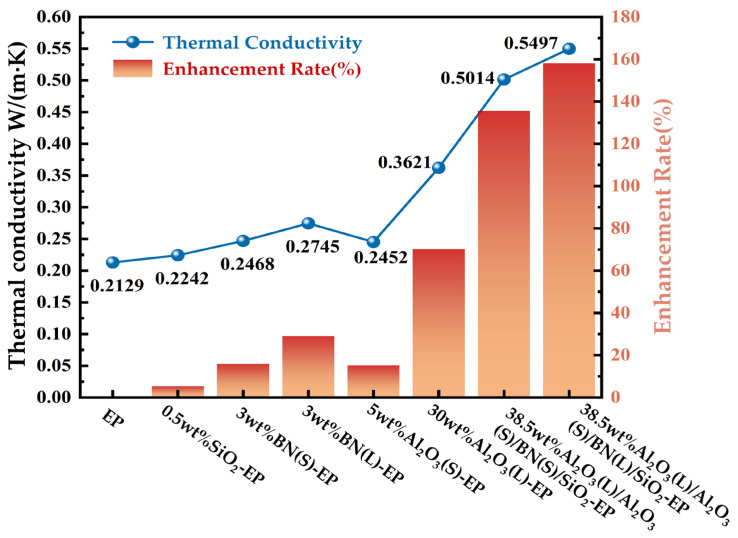
Thermal conductivity measurement values and percentage improvement of epoxy resin composite materials.

As shown in Figure 8, the incorporation of either single-particle fillers or multiscale hybrid fillers increases the thermal conductivity of EP compared with neat epoxy resin.

The experimentally measured thermal conductivity values show a variation trend consistent with the simulation results in Figure 4. This agreement indicates that the established simulation model can reasonably predict the thermal conductivity behavior of the composites.

According to Figure 8, the thermal conductivity values of the investigated systems follow the order: EP < 0.5 wt%SiO_2_-EP < 5 wt%Al_2_O_3_(S)-EP < 3 wt%BN(S)-EP < 3 wt%BN(L)-EP < 30 wt%Al_2_O_3_(L)-EP < 38.5 wt%Al_2_O_3_(L)/Al_2_O_3_(S)/BN(S)/SiO_2_-EP < 38.5 wt%Al_2_O_3_(L)/Al_2_O_3_(S)/BN(L)/SiO_2_-EP. Among all the investigated formulations, 38.5 wt%Al_2_O_3_(L)/Al_2_O_3_(S)/BN(L)/SiO_2_-EP and 38.5 wt%Al_2_O_3_(L)/Al_2_O_3_(S)/BN(S)/SiO_2_-EP, exhibit significantly higher thermal conductivity than the single-filler systems.

#### 4.2.2. Discussion of Thermal Conductivity Results

The superior thermal conductivity of the multiscale hybrid-filler systems can be attributed to the synergistic thermal-conduction network constructed by fillers with different sizes and morphologies. At high filler loading, large Al_2_O_3_ particles form the primary thermally conductive framework, while small Al_2_O_3_ particles fill the gaps between adjacent large particles and improve the packing density of the system.

However, the point-to-point contact between spherical fillers is still relatively inefficient for heat transfer. The introduction of a small amount of platelet BN increases the effective contact area between neighboring particles and bridges adjacent Al_2_O_3_ particles, thereby promoting the formation of a more continuous heat-conduction pathway. This bridging effect improves the heat-transfer efficiency of the composite and contributes significantly to the enhancement of thermal conductivity [23].

**Figure 9:**
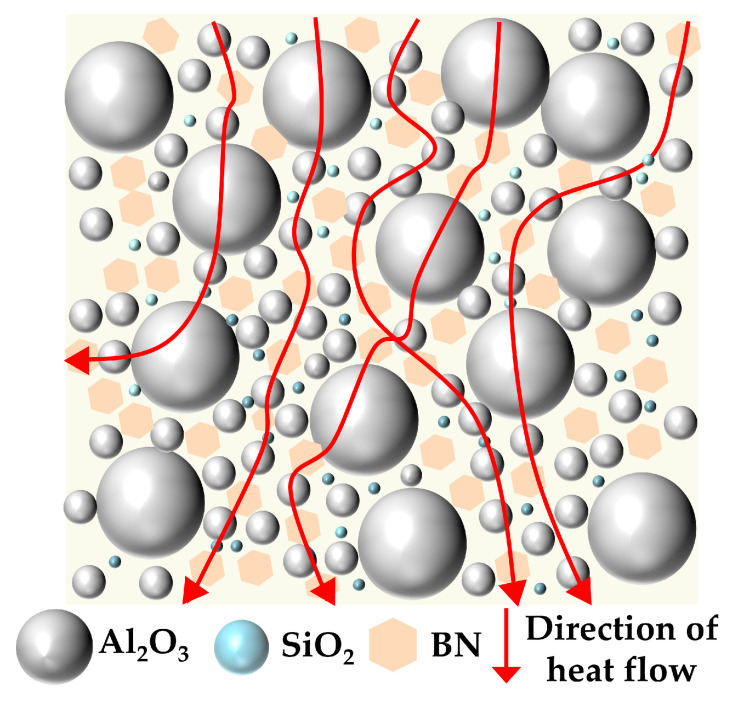
Schematic diagram of the internal thermal conductivity network of epoxy resin composites.

In addition, the incorporation of low-content nano-SiO_2_ is beneficial for improving interfacial compatibility and reducing interfacial defects in the composite [24]. As a result, phonon scattering at the filler–matrix interface can be suppressed to some extent, which further enhances the overall thermal conductivity. The multiscale thermal-conduction network formed in the composite is schematically illustrated in Figure 9.

It should also be noted that the single-filler systems shown in Figure 8 were selected as representative formulations, rather than as a strict equal-loading comparison among all filler types. Under the present experimental conditions, a direct comparison at the same high filler loading (38.5 wt%) was not feasible for all fillers. Compared with spherical Al_2_O_3_ particles, platelet BN and nano-SiO_2_ particles possess higher specific surface area, lower packing efficiency, and stronger particle–particle interactions, which result in a sharp increase in viscosity and severe dispersion difficulty at high loading. Therefore, under the current laboratory mixing conditions, it was difficult to prepare uniform and testable BN/EP and SiO_2_/EP composites at 38.5 wt%.

### 4.3. Dielectric Properties

#### 4.3.1. Dielectric Property Results

**Figure 10:**
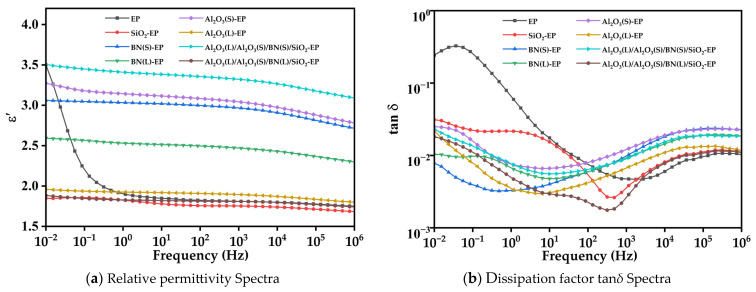
Relative permittivity and dissipation factor tan*δ* spectra of epoxy resin composites.

Figure 10 shows the relative permittivity (*ε′*) and dissipation factor tan*δ* spectra of neat EP and EP composites over the frequency range from 10^−2^ to 10^6^ Hz. As shown in Figure 10a, the *ε′* values of all samples decrease monotonically with increasing frequency. In the low-frequency region (<1 Hz), neat EP exhibits a more obvious increase in *ε′* than most of the composite systems. Within the frequency range from 1 Hz to 10^6^ Hz, the *ε′* values of the composites filled with single micron-sized particles are all higher than that of neat EP, and the order is Al_2_O_3_(S)-EP > BN(S)-EP > BN(L)-EP > Al_2_O_3_(L)-EP. Among all the investigated systems, the 0.5 wt%nano-SiO_2_-EP composite exhibits the lowest *ε′* value.

For the hybrid system Al_2_O_3_(L)/Al_2_O_3_(S)/BN(L)/SiO_2_-EP, the *ε′* value is lower than that of neat EP in the low-frequency region and remains close to that of neat EP in the frequency range from 1 to 10^6^ Hz. By contrast, the *ε′* value of Al_2_O_3_(L)/Al_2_O_3_(S)/BN(S)/SiO_2_-EP remains the highest over the entire frequency range from 10^−2^ to 10^6^ Hz.

As shown in Figure 10b, the tan*δ* spectra of all materials first decrease and then increase with increasing frequency. In the low-frequency region, the tan*δ* values of all composites are lower than that of neat EP, whereas in the high-frequency region, the tan*δ* values of all composites become higher than that of neat EP.

#### 4.3.2. Discussion of Dielectric Properties

The monotonic decrease in *ε′* with frequency indicates that the polarization processes in all systems gradually fail to follow the alternating electric field as the frequency increases. The more pronounced low-frequency increase in *ε′* for neat EP suggests that its dielectric response in this region is mainly governed by electrode polarization and conduction-related space-charge accumulation. After filler incorporation, the interfacial regions and trap sites formed in the composites suppress charge injection and carrier migration, thereby weakening the low-frequency rise in *ε′*.

Among all the investigated systems, the nano-SiO_2_-EP composite exhibits the lowest *ε′*, because at 0.5 wt% loading, the interfacial regions formed by nano-SiO_2_ restrict polymer-chain motion and dipole orientation [25]. For the hybrid system Al_2_O_3_(L)/Al_2_O_3_(S)/BN(L)/SiO_2_-EP, the *ε′* value is lower than that of neat EP in the low-frequency region. It remains close to that of neat EP between 1 and 10^6^ Hz. This may be due to the interfacial constraint effect of nano-SiO_2_ and the preferential parallel orientation of micron-sized BN platelets. Both factors help suppress dipole orientational polarization and restrict molecular-chain mobility. By contrast, the *ε′* value of Al_2_O_3_(L)/Al_2_O_3_(S)/BN(S)/SiO_2_-EP remains the highest over the whole frequency range from 10^−2^ to 10^6^ Hz. This may be related to the larger specific interfacial area introduced by the smaller BN particles. The larger interfacial area enhances interfacial polarization and results in a higher overall dielectric response [26,27,28].

The tan*δ* behavior originates from two aspects: one is the loss caused by electrical conduction, and the other is the loss generated by relaxation polarization. In the low-frequency region, the dipoles in the epoxy resin matrix can follow the variation in the electric field frequency, so the loss caused by polarization is small, and the dielectric loss is mainly attributed to electrical conduction. With the increase in frequency, some dipoles with larger functional groups cannot polarize in time, leading to a decrease in dielectric constant and an increase in dielectric loss [29].

Therefore, the lower tan*δ* values of all composites in the low-frequency region can be attributed to the deep traps formed in the interfacial regions between the fillers and the epoxy matrix. These deep traps capture mobile charge carriers and suppress carrier transport, thereby reducing the conduction-related loss [21]. In contrast, the higher tan*δ* values of the composites in the high-frequency region are mainly associated with the additional relaxation processes introduced by the fillers. These include the dipolar response from the interfacial regions and the relaxation of polar groups on the filler surfaces, which together enhance the dielectric loss at higher frequencies.

### 4.4. Breakdown Characteristics

#### 4.4.1. Breakdown Strength Results

**Figure 11:**
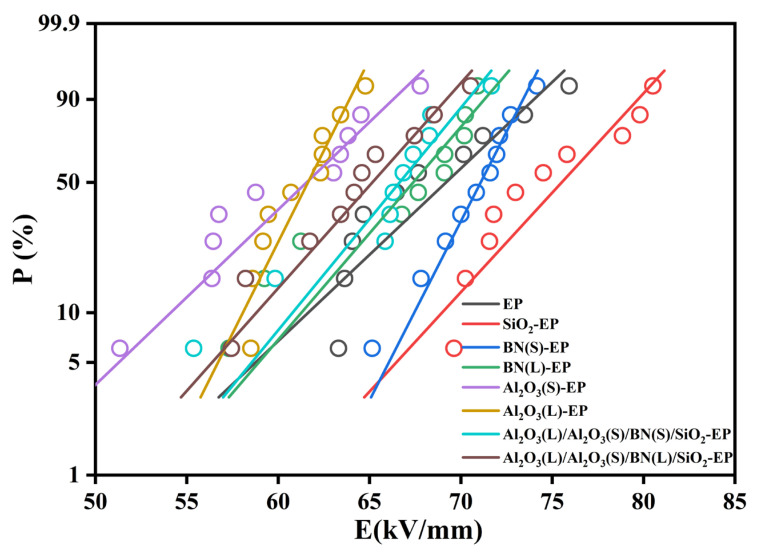
Breakdown Weibull distribution map of epoxy composites.

In the power frequency breakdown test, the applied voltage is high, whereas the action time is short, and the heat generation of the sample is limited. Under such conditions, it is difficult to accumulate a critical temperature rise. Therefore, the breakdown occurring under this condition is considered an electrical breakdown. Breakdown strength is an important performance index for epoxy resins used as the main insulation for stator bars in large and medium-sized generators. This study was carried out using a self-built test platform. Figure 11 shows the Weibull plots of electrical breakdown for neat EP and the particle-filled epoxy composites. Table 5 shows the characteristic breakdown field strength (*E*_0_) and improvement percentage of the Weibull distribution at a breakdown probability of 63.2%.

As shown in Figure 11 and Table 5, the 0.5 wt%SiO_2_-EP sample exhibits the highest characteristic breakdown field strength, reaching 76.43 kV/mm, which is 8.99% higher than that of neat EP. Among the single-filler systems, BN(S)-EP and BN(L)-EP show better insulation performance than neat EP, with the breakdown field strength increasing by 2.21% and 2.87%, respectively. In contrast, Al_2_O_3_(S)-EP and Al_2_O_3_(L)-EP show reduced breakdown strength, with decreases of 2.71% and 10.99%, respectively, relative to neat EP. Among the hybrid systems, Al_2_O_3_(L)/Al_2_O_3_(S)/BN(L)/SiO_2_-EP exhibits the lowest breakdown field strength of 65.99 kV/mm, which is 5.89% lower than that of neat EP.

#### 4.4.2. Discussion of Breakdown Strength

The superior insulation performance of 0.5 wt%SiO_2_-EP can be attributed to the combined effects of dielectric matching and interfacial trap regulation. First, the relative permittivity of SiO_2_ is closest to that of epoxy resin, which significantly reduces the degree of electric field distortion inside the composite material, rendering the electric field distribution more uniform, decreasing the probability of breakdown induced by local electric field concentration, and thus enhancing the insulation performance of the material. Second, the large interfacial area between nano-sized SiO_2_ particles and EP creates more deep trap sites, which not only raises the energy level and density of deep traps, but also effectively suppresses carrier injection and space charge accumulation, delays the formation and expansion of breakdown channels, and thus improves the breakdown voltage [30].

For the single-filler systems, the improvement in the breakdown strength of BN(S)-EP and BN(L)-EP may be related to the close interaction between low-content BN particles and the epoxy matrix. On the one hand, BN particles can increase the barrier height for electron transport and inhibit the initial injection of electrons. On the other hand, they can fill or reduce micro-defects in the matrix, thereby hindering the formation of breakdown paths [31].

By contrast, the reduced breakdown strength of Al_2_O_3_(S)-EP and Al_2_O_3_(L)-EP is likely due to dielectric mismatch and particle agglomeration. The higher permittivity of Al_2_O_3_ relative to the epoxy matrix causes local electric-field distortion and a non-uniform field distribution. In addition, the high surface energy of Al_2_O_3_ promotes agglomeration, and these clusters can serve as channels for electrical tree growth, thereby degrading the insulation performance [21].

For the hybrid system Al_2_O_3_(L)/Al_2_O_3_(S)/BN(L)-EP, the reduced breakdown strength is mainly attributed to the combined effects of high filler loading and intensified interfacial field distortion. At high overall filler content, the number of internal defects in the composite increases, making it easier for initial electrons to trigger secondary electron generation and thus lowering the breakdown threshold [32]. Meanwhile, the dielectric-constant difference between the epoxy matrix and the fillers further intensifies interfacial electric-field distortion, leading to a highly non-uniform electric-field distribution inside the material and consequently a decline in breakdown performance.

## 5. Conclusions

To improve the thermal conductivity of epoxy resin, this study systematically investigated the effects of Al_2_O_3_, BN, and SiO_2_ on the thermal conductivity and insulation properties of EP composites through a multi-scale and multi-shape particle compounding strategy, integrated with finite element simulation and experimental testing. The results indicate that:

(1) By compounding spherical Al_2_O_3_ with large and small particle sizes, Platelet BN, and spherical SiO_2_, the thermal conductivity of the composite material is increased to 0.5497 W/(m·K) at a total filler content of 38.5 wt%, which is 158.2% higher than that of pure EP. The underlying mechanism is that multi-scale particles synergistically construct a continuous thermally conductive network structure. That is, large particle size Al_2_O_3_ forms a thermal conductivity framework, small particle size Al_2_O_3_ fills the gaps, platelet BN bridges particles to increase the contact area, and nano-SiO_2_ enhances interfacial compatibility and reduces phonon scattering.
(2) Compared to pure EP, the SiO_2_-EP and Al_2_O_3_(L)/Al_2_O_3_(S)/BN(L)/SiO_2_-EP composites exhibit reduced *ε*′ values. Their tan*δ* is lower in the low-frequency region and comparable to that of pure EP at high frequencies. This behavior is attributed to the interfacial regions formed between the micro/nano particles and the epoxy matrix, which restrict the segmental motion of polymer chains and dipole polarization, thereby further decreasing tan*δ.*
(3) The SiO_2_-EP composite exhibits the highest breakdown strength of 76.43 kV/mm, which can be attributed to the more uniform distribution of smaller particles that minimizes internal electric field distortion. In contrast, the Al_2_O_3_(L)/Al_2_O_3_(S)/BN(L)/SiO_2_-EP composite shows a reduced breakdown strength of 65.99 kV/mm. This reduction is attributed to the high filler loading of multiple particles, which introduces more internal defects and exacerbates electric field distortion at the particle-matrix interfaces. Nevertheless, this value still satisfies the basic insulation requirements for generator main insulation.

Overall, this work demonstrates that a simulation-guided multiscale and multishape hybrid filler strategy is an effective route for improving the thermal conductivity of epoxy composites while maintaining acceptable insulating performance, and provides useful guidance for the design of thermally conductive insulating materials for generator applications.

## 6. Outlook

In future work, the dielectric properties of the composites under elevated-temperature conditions should be further investigated to better evaluate their practical applicability under electrothermal coupling conditions. In addition, the present simulation model can be further improved by considering interfacial thermal resistance, non-ideal particle–matrix contact, and more realistic multiscale interface effects, which would help establish a more accurate relationship between the simulated and experimental thermal-transport behavior of epoxy composites.

## Figures and Tables

**Table 1:** Experimental formulations of the epoxy composites prepared in this work.

Group	Sample Name	Mass Fraction wt%					
		EP	SiO_2_ (30 nm)	BN (50 nm)	BN (1 μm)	Al_2_O_3_ (1 μm)	Al_2_O_3_ (10 μm)
G1	EP	100					
G2	0.5 wt%SiO_2_-EP	99.5	0.5				
G3	3 wt%BN(S)-EP	97		3			
G4	3 wt%BN(L)-EP	97			3		
G5	5 wt%Al_2_O_3_(S)-EP	95				5	
G6	30 wt%Al_2_O_3_(L)-EP	70					30
G7	38.5 wt%Al_2_O_3_(L)/Al_2_O_3_(S)/ BN(S)/SiO_2_-EP	61.5	0.5	3		5	30
G8	38.5 wt%Al_2_O_3_(L)/Al_2_O_3_(S)/ BN(L)/SiO_2_-EP	61.5	0.5		3	5	30

**Table 2:** Material parameters.

Material	Specific Heat Capacity/(J/(Kg·K))	Density/(kg/m^3^)	Thermal Conductivity/(W/(m·K))	Relative Permittivity
Epoxy resin	1150	1200	0.2	3.87
Al_2_O_3_ particles	800	3970	30	3.9
BN particles	700	2280	300	4.8
SiO_2_ particles	750	2000	1	9.8

**Table 3:** Simulation cases used in the finite-element model.

Group	Sample Name	Mass Fraction wt%					
		EP	SiO_2_ (30 nm)	BN (50 nm)	BN (1 μm)	Al_2_O_3_ (1 μm)	Al_2_O_3_ (10 μm)
S1	EP	100					
S2	0.5 wt%SiO_2_-EP	99.5	0.5				
S3	3 wt%BN(S)-EP	97		3			
S4	3 wt%BN(L)-EP	97			3		
S5	5 wt%Al_2_O_3_(S)-EP	95				5	
S6	30 wt%Al_2_O_3_(L)-EP	70					30
S7	38 wt%Al_2_O_3_(L)/Al_2_O_3_(S)/ BN(L)-EP	62			3	5	30

**Table 4:** Effect of filler thermal conductivity variation on the predicted effective thermal conductivity of the composite.

Filler	Thermal Conductivity Input (W·m^−1^·K^−1^)	Effective Thermal Conductivity of Composite (W·m^−1^·K^−1^)	Relative Variation
Al_2_O_3_	24	0.3492	−1.2
Al_2_O_3_	30 (reference)	0.3536	0
Al_2_O_3_	36	0.3568	0.90
BN	240	0.2522	−0.55
BN	300 (reference)	0.2536	0
BN	360	0.2552	0.63
SiO_2_	0.8	0.21503	−0.07
SiO_2_	1 (reference)	0.21650	0
SiO_2_	1.2	0.21686	0.02

**Table 5:** Breakdown field strength of each group of epoxy composites.

Group	Sample Name	Breakdown Field Strength *E*_0_	Improvement Percentage Compared to EP/%
G1	EP	70.12	0
G2	0.5 wt%SiO_2_-EP	76.43	8.99
G3	3 wt%BN(S)-EP	71.67	2.21
G4	3 wt%BN(L)-EP	72.13	2.87
G5	5 wt%Al_2_O_3_(S)-EP	68.22	−2.71
G6	30 wt%Al_2_O_3_(L)-EP	62.41	−10.99
G7	38.5 wt%Al_2_O_3_(L)/Al_2_O_3_(S)/BN(S)/SiO_2_-EP	67.45	−3.81
G8	38.5 wt%Al_2_O_3_(L)/Al_2_O_3_(S)/BN(L)/SiO_2_-EP	65.99	−5.89

## Data Availability

The raw data supporting the conclusions of this article will be made available by the authors on request.
